# The New High-Pressure Phases of Nitrogen-Rich Ag–N Compounds

**DOI:** 10.3390/ma15144986

**Published:** 2022-07-18

**Authors:** Ran Liu, Dan Xu, Zhen Yao, Shifeng Niu, Bingbing Liu

**Affiliations:** 1 State Key Laboratory of Superhard Materials, College of Physics, Jilin University, Changchun 130012, China; liuran@jlu.edu.cn (R.L.); xudan@jlu.edu.cn (D.X.); 2 School of Physics and Engineering, Henan University of Science and Technology, Luoyang 471023, China

**Keywords:** polymeric nitrogen, high-energy density, high-pressure

## Abstract

The high-pressure phase diagram of Ag–N compounds is enriched by proposing three stable high-pressure phases (P4/mmm-AgN_2_, P1-AgN_7_ and P-1-AgN_7_) and two metastable high-pressure phases (P-1-AgN_4_ and P-1-AgN_8_). The novel N_7_ rings and N_20_ rings are firstly found in the folded layer structure of P-1-AgN_7_. The electronic structure properties of predicted five structures are studied by the calculations of the band structure and DOS. The analyses of ELF and Bader charge show that the strong N–N covalent bond interaction and the weak Ag–N ionic bond interaction constitute the stable mechanism of Ag–N compounds. The charge transfer between the Ag and N atoms plays an important role for the structural stability. Moreover, the P-1-AgN_7_ and P-1-AgN_8_ with the high-energy density and excellent detonation properties are potential candidates for new high-energy density species.

## 1. Introduction

Due to the significant energy differences between the N–N bond, N=N bond and N≡N bond, polymeric nitrogen with the single/double bond structure is the potential high-energy-density materials (HEDMs). Moreover, the decomposition product of polymeric nitrogen is the clean diatomic nitrogen gas (N_2_). Thus, the polymeric nitrogen can be used as the environmentally friendly HEDM. Many efforts have been performed for exploring the novel polymeric nitrogen structures, such as the chain-shaped structures (ch, Cmcm, PP) [1,2], the layered structures (A7, BP-N, LP-N, LB-N) [1,3,4,5], the caged structures (N_10_) [6], the networked structures (cg-N, rcg-N, P-42_1_m, P2_1_2_1_2_1_, P2_1_/m, C2/c, P2_1_2_1_2_1_-500, Pnnm, P2_1_, CW) [1,7,8,9] and the molecular crystal structures (N_2_-N_6_, N_6_, N_8_, 2N_5_) [10,11,12,13]. Up to now, the cg-N, BP-N, LP-N and HLP-N have been successfully synthesized at (110 GPa, 2000 K), (146 GPa, 2200 K), (150 GPa, 3000 K) and (244 GPa, 3300 K), respectively. The study of decomposition shows that the cg-N, BP-N, LP-N and HLP-N can be quenched down to 42 GPa, 48 GPa, 52 GPa and 66 GPa, respectively [14,15,16,17,18]. Clearly, the harsh synthesis conditions (*P* > 100 GPa, T > 2000 K) and the low stability of polymeric nitrogen limit its application.

Recent studies show that introducing an impurity element (M) into the pure nitrogen structure can induce the novel polynitrogen structures, which may exhibit the excellent properties, such as the mild synthesis conditions, high stability, etc. A series of polynitrogen structures has been reported in theoretical studies. Typically, the novel N_4_ ring is reported for P4/mmm-MnN_4_, Cm-Al_2_N_7_, P-1-Na_2_N_8_, P2_1_/c-Li_2_N_4_, C2/m-MgN_4_ and Immm-AlN_4_ compounds [19,20,21,22]. Especially, the regular N_4_ rings results in a superhard property of P4/mmm-MnN_4_ and Cm-Al_2_N_7_. The N_5_ ring structures are found in the M_2_N_5_ (M = Na) and MN_5_ (M = Li, Na, Rb, Cs, Ca, Sr, Ba, Cu) compounds [23,24,25,26,27,28,29]. More interestingly, the double-, triple- and quadruple-N_5_ ring structures are found in MN_10_ (M = Be, Mg, Ba), MN_15_ (M = Al, Ga, Sc, Y) and HfN_20_ compounds, respectively [30,31,32,33]. Among these reported structures, the P2_1_-LiN_5_, Cm-NaN_5_, Pc-RbN_5_ and P-1-BaN_10_ are stable with the pressure larger than 9.9 GPa, 20 GPa, 30 GPa and 12 GPa, respectively. The Fdd2-BeN_10_ and Fdd2-MgN_10_ compounds are possibly synthesized at relatively low pressures (around 28 GPa for BeN_10_ and 12 GPa for MgN_10_) and can be preserved under ambient pressure. The good gravimetric energy density of Fdd2-BeN_10_ (5.39 kJ/g), Fdd2-MgN_10_ (3.48 kJ/g) and Cc-AlN_15_ (5.31 kJ/g) makes them the potential (HEDMs). The novel N_6_ ring structures are found in MN_3_ (M = Cs, Ca, Sr, K, Mg) [26,27,28,34,35,36,37,38] and MN_6_ (M = W) compounds [39,40]. Among them, the P-1-MgN_3_ phase with the N_6_ ring structure is recoverable at ambient pressure [36]. The superhard R-3m-WN_6_ remains dynamically stable at ambient conditions [40]. The N-chain structures are found in MN_4_ (M = Be, Cd, Fe, Gd, Re, Os, W, Ru, Zn) [41,42,43,44,45,46,47,48], GdN_6_ [45], ReN_8_ [49] and HfN_10_ compounds [50]. Among them, P-1-BeN_4_, γ-P-1-BeN_4_ and δ-P-1-BeN_4_ with the N-chain structures can be synthesized under pressures of 25.4, 20.8 and 27.4 GPa, respectively, which is greatly lower than 110 GPa for synthesizing the cg-N. The analysis of dynamical and thermal stability shows that the P-1-GdN_6_ can be recovered to ambient conditions upon synthesis under compression. The Immm-HfN_10_ is discovered to be stable at moderate pressure above 23 GPa and can be preserved as a metastable phase at ambient pressure. The novel N_18_-ring, N_6_ + N_10_-ring, N_10_-ring and N_18_-ring layered structures are found in P6/mcc-K_2_N_16_, C2/m-BaN_6_, P2_1_/c-BeN_4_ and P-31c-CoN_8_ compounds, respectively [31,51,52,53]. Moreover, the N_14_-ring band-shape structure is the first reported for P-1-CoN_10_ [53]. The P-31c-CoN_8_ (6.14 kJ/g) and P-1-CoN_10_ (5.18 kJ/g) with high-energy density can be quenched down to ambient conditions. The three-dimensional network structures are found in the C2/m-CdN_6_, I4_1_/a-HeN_4_, R-3m-HeN_6_, P63/m-HeN_10_ and C2/m-HeN_22_ [43,54,55]. The C2/m-CdN_6_ and C2/m-HeN_22_ with respectively high-energy-density values of 3.82 kJ/g and 10.44 kJ/g may be quenchable to ambient pressure. In the experiment, the cyclo-N_5_ ring in LiN_5_, NaN_5_ and CsN_5_ compounds are synthesized at 45, 52 and 65 GPa, respectively [56,57,58]. The CsN_5_ and LiN_5_ can be quenched down to 18 GPa and ambient pressure, respectively. The armchair-like hexazine N_6_ ring in R-3m-WN_6_ is synthesized with pressure larger than 126 GPa [59]. The N-chain structure in Ibam-MgN_4_ and FeN_4_ are synthesized at 50 and 180 GPa, respectively [60,61]. As the review above, we know that the introduced impurity element can induce the novel polymeric nitrogen structures, which may exhibit more prominent properties than the pure polymeric nitrogen structures, such as the milder synthesis pressure and the higher stability.

Silver nitrides have received much attention for their outstanding chemical and physical properties, such as the energetic explosive, propulsion application, gas generators, photographic materials, etc. [62,63,64]. Recently, the armchair–antiarmchair N-chain and N_5_ ring structures are severally reported for AgN_3_ and AgN_5_/AgN_6_ compounds [65,66]. Beyond that, no other new silver nitrides with the high-pressure polymeric structures have been reported. Thus, a detailed high-pressure study that considers the different stoichiometry in silver nitrides is necessary for exploring new polynitrogen polymeric structures.

## 2. Computation Details

The structural research has been performed by the particle swarm optimization methodology implemented in the CALYPSO structure prediction method [67]. The structure optimizations and property calculations have been carried out by the Vienna ab initio simulation package (VASP) code [68]. The generalized gradient approximation (GGA) with the Perdew–Burke–Ernzerhof (PBE) exchange–correlation function has been employed for the first-principles calculations [69,70,71]. The 4d^10^5s^1^ and 2s^2^2p^3^ are treated as the valence electrons of Ag and N atoms, respectively. In order to ensure that the enthalpy is converged to less than 1 meV/atom, the cutoff energy of Projector Augmented Wave (PAW) pseudopotential and the Monkhorst–Pack k-mesh density are severally set to 520 eV and 2π × 0.03 Å^−1^ in the calculation. The ΔH^f^ of each Ag–N structure is calculated by using the following equation: ΔH^f^ (AgN_x_) = [H(AgN_x_) − H(AgN) − (x − 1) H(N)]/(1 + x). The most stable structures of AgN (Abma-phase) and N (P2/c- and cg-phases) are chosen as the reference structures in their corresponding stable pressure range. The phonon frequencies have been calculated by using the finite displacement approach through the PHONOPY code [72]. The 2 × 2 × 2 supercell with the lattice size of about 10 Å is constructed in the calculation of phonon. The dissociation energies are calculated by considering the following decomposition paths: AgN_x_ → Ag + x/2N_2_. The P6_3_/mmc phase of Ag and Pa $\bar{3}$ phase of N_2_ are the decomposition productions, respectively. The detonation velocity and detonation pressure have been calculated by using the Kamlet–Jacobs simi-empirical equation: V_d_ = 1.01(NM^0.5^E_d_^0.5^)^0.5^(1 + 1.30ρ) and P_d_ = 15.58ρ^2^NM^0.5^E_d_^0.5^. N represents the moles of gas per gram of AgNx, M represents the average molar mass of gas products, E_d_ is the detonation chemical energy, and ρ is the mass density.

## 3. Results and Discussion

**Figure 1:**
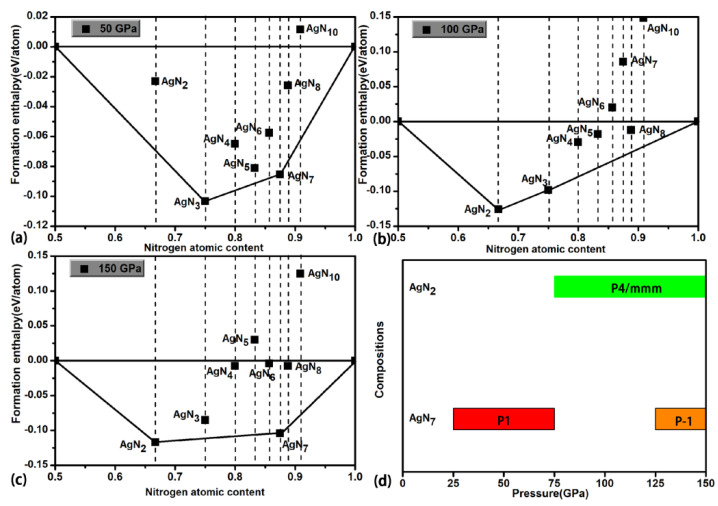
The formation enthalpies of Ag-N phases with respect to the Abma-AgN phase and nitrogen solids at different pressures (**a**–**c**). (**d**) shows the phase diagram of AgN_2_ and AgN_7_.

**Figure 2:**
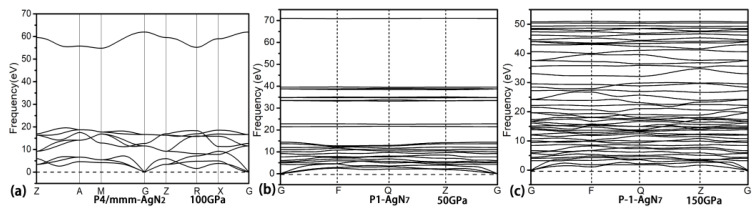
The phonon dispersion of P4/mmm-AgN_2_ at 100 GPa (**a**), the P1-AgN_7_ at 50 GPa (**b**) and P-1-AgN_7_ at 150 GPa (**c**).

**Figure 3:**
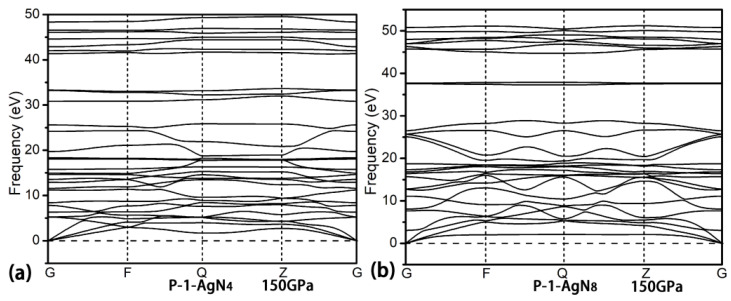
The phonon dispersion of P-1-AgN_4_ at 150 GPa (**a**) and P-1-AgN_8_ at 150 GPa (**b**).

Eight stoichiometries of AgN_x_ (x = 2, 3, 4, 5, 6, 7, 8, 10) are considered in the structural research with the simulation cells containing 1, 2 and 4 formula units (f.u.). The prediction for each stoichiometry is carried out at three pressures (50, 100 and 150 GPa). As shown in Figure 1a–c, the formation enthalpies (ΔH^f^) of Ag–N compounds are presented in the thermodynamic convex hull. The solid squares on the convex hull are the thermodynamically stable phases, while the ones that deviate from the convex hull are the metastable/unstable phases. For the AgN_2_ stoichiometry, we found the thermodynamically stable P4/mmm phase at 100 and 150 GPa. At 50 and 100 GPa, the reported P-1-AgN_3_ in Ref. [65] is also found in this work. For the AgN_7_ stoichiometry, the thermodynamically stable P1 and P-1 phases are found at 50 GPa and 100/150 GPa, respectively. No thermodynamically stable phases are found for the rest of the stoichiometries (AgN_4_, AgN_5_, AgN_6_, AgN_8_ and AgN_10_). For the presented high-pressure phase diagrams of AgN_2_ and AgN_7_ in Figure 1d, we can see that the P4/mmm-AgN_2_ is thermodynamically stable in the pressure region of (75–150 GPa). The P1-AgN_7_ and P-1-AgN_7_ are thermodynamically stable in the pressure ranges of (25–75 GPa) and (125–150 GPa), respectively. The dynamical stability of AgN_2_ and AgN_7_ are further evaluated by the phonon dispersion. As shown in Figure 2, no imaginary frequency is found throughout the Brillouin zone, indicating that the P4/mmm-AgN_2_, P1-AgN_7_ and P-1-AgN_7_ are dynamically stable at 100, 50 and 150 GPa, respectively. Interestingly, the presented phonon dispersion curves in Figure 3 show that the P-1-AgN_4_ and P-1-AgN_8_ are dynamically stable at 150 GPa, indicating that they are the metastable phases. Moreover, the mechanical stabilities of P4/mmm-AgN_2_, P1-AgN_7_, P-1-AgN_7_, P-1-AgN_4_ and P-1-AgN_8_ are evaluated by the calculation of elastic constants (Table 1). According to the mechanical stability criteria of tetragonal structure of (C_11_ > |C_12_|, 2C_13_^2^ < C_33_ (C_11_ + C_12_), C_44_ > 0), we know that the tetragonal P4/mmm-AgN_2_ is mechanically stable. The mechanical stability criteria of monoclinic structure are shown as follows [73]:

C_11_ > 0, C_22_ > 0, C_33_ > 0, C_44_ > 0, C_55_ > 0, C_66_ > 0, [C_11_ + C_22_ + C_33_ + 2 (C_12_ + C_13_ + C_23_)] > 0, C_33_C_55_-C_35_^2^ > 0, C_44_C_66_-C_46_^2^ > 0, C_22_ + C_33_-2C_23_ > 0, C_22_ (C_33_C_55_-C_35_^2^) + 2C_23_C_25_C_35_-(C_23_^2^) C_55_-(C_25_^2^) C_33_ > 0, 2 [C_15_C_25_ (C_33_C_12_-C_13_C_23_) + C_15_C_35_ (C_22_C_13_-C_12_C_23_) + C_25_C_35_ (C_11_C_23_-C_12_C_13_)]-[C_15_^2^ (C_22_C_33_-C_23_^2^) + C_25_C_25_ (C_11_C_33_-C_13_^2^) + C_35_C_35_ (C_11_C_22_-C_12_^2^)] + C_55_g > 0.

We can see that the elastic tensors Cij of P1-AgN_7_, P-1-AgN_7_, P-1-AgN_4_ and P-1-AgN_8_ satisfy the criteria, indicating that they possess the mechanical stability. Thus, we proposed three stable high-pressure phases (P4/mmm-AgN_2_, P1-AgN_7_ and P-1-AgN_7_) and two metastable high-pressure phases (P-1-AgN_4_ and P-1-AgN_8_) by the structural prediction method.

**Figure 4:**
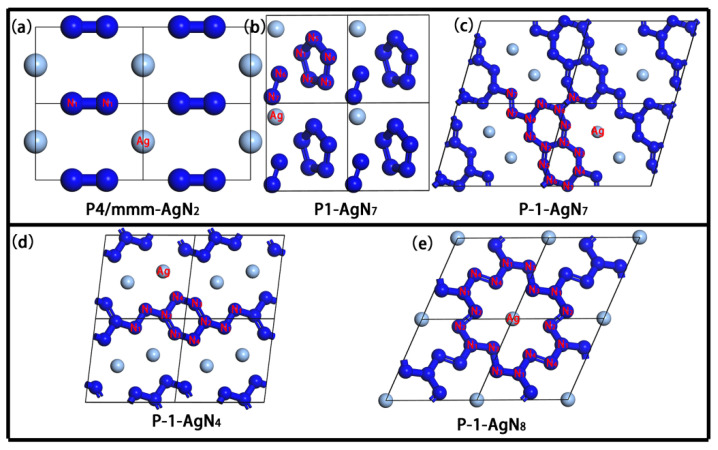
The 2 × 2 × 2 supercell structures of Ag-N compounds: P4/mmm-AgN_2_ (**a**), P1-AgN_7_ (**b**), P-1-AgN_7_ (**c**), P-1-AgN_4_ (**d**) and P-1-AgN_8_ (**e**).

The crystal structures of P4/mmm-AgN_2_, P1-AgN_7_, P-1-AgN_7_, P-1-AgN_4_ and P-1-AgN_8_ are presented in Figure 4. In P4/mmm-AgN_2_, the polymeric N-structure unit is the dumbbell-shaped N_2_ structure, which is composed of two equivalent nitrogen atoms. At 100 GPa, the bond length of N_1_-N_1_ is 1.165 Å. For the P1-AgN_7_ presented in Figure 4b, one unit cell contains one dumbbell-shaped N_2_ structure and one N_5_ ring structure. The N_5_ ring structure is composed of five inequitable nitrogen atoms (N_1_->N_5_), while the dumbbell-shaped N_2_ structure is composed of two inequitable nitrogen atoms (N_6_-N_7_). At 50 GPa, the bond lengths of N_1_-N_5_, N_2_-N_3_, N_3_-N_4_, N_4_-N_5_, N_5_-N_1_ and N_6_-N_7_ are 1.296 Å, 1.309 Å, 1.296 Å, 1.301 Å, 1.307 Å and 1.114 Å, respectively. The P-1-AgN_7_ is the folded layer structure, which is constituted by the N_20_ ring and two fused N_7_ rings. At 150 GPa, the bond lengths of ten N–N bonds (N_1_-N_4_, N_4_-N_3_, N_3_-N_2_, N_2_-N_2_, N_2_-N_5_, N_5_-N_6_, N_6_-N_1_, N_6_-N_6_, N_4_-N_7_ and N_7_-N_7_) that are constructed by seven inequitable nitrogen atoms (N_1_->N_7_) are 1.286 Å, 1.315 Å, 1.270 Å, 1.286 Å, 1.274 Å, 1.282 Å, 1.254 Å, 1.318 Å, 1.277 Å and 1.261 Å, respectively. Up to now, the reported N-rings in the polynitrogen structures are the N_5_, N_6_, N_10_, N_12_, N_14_ and N_18_ rings [51,52,53,54]. As the construction unit of the layer structure, the novel N_7_ rings and N_20_ rings are firstly reported for this work. The P-1-AgN_4_ is the 1-D chain structure, which is constructed by the alternate N_2_ and N_6_ ring. At 150 GPa, the bond lengths of five N–N bonds (N_1_-N_1_, N_1_-N_2_, N_2_-N_3_, N_3_-N_4_ and N_4_-N_2_) that are constructed by four inequitable nitrogen atoms (N_1_->N_4_) are 1.266 Å, 1.282 Å, 1.300 Å, 1.281 Å and 1.306 Å, respectively. The P-1-AgN_8_ is the layer structure, which is constructed by the fused N_18_ ring structure. At 150 GPa, the bond lengths of five N–N bonds (N_1_-N_4_, N_4_-N_4_, N_1_-N_2_, N_2_-N_2_ and N_1_-N_3_,) that are constructed by four inequitable nitrogen atoms (N_1_->N_4_) are 1.271 Å, 1.257 Å, 1.272 Å, 1.269 Å and 1.280 Å, respectively.

**Figure 5:**
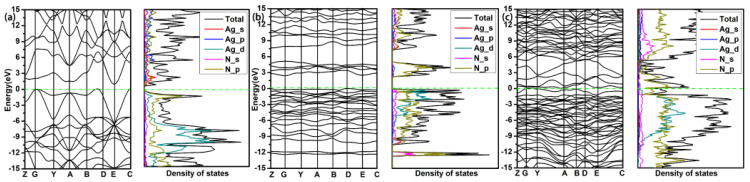
Band structures and projected density of states of P4/mmm-AgN_2_ at 100 GPa (**a**), P1-AgN_7_ at 50 GPa (**b**), P-1-AgN_7_ at 150 GPa (**c**).

**Figure 6:**
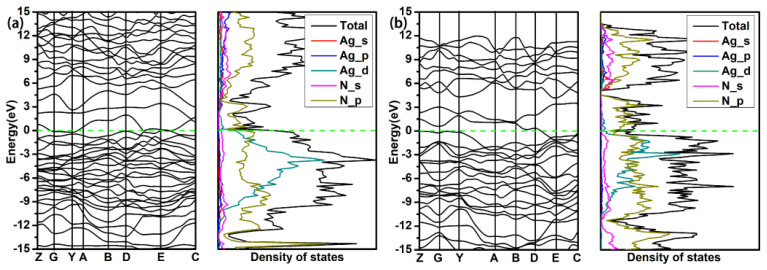
Band structures and projected density of states of P-1-AgN_4_ (**a**) and P-1-AgN_8_ (**b**) at 150 GPa.

The electronic structural properties including the band structure, the density of states (DOS), the electronic local function (ELF) and the Bader charge transfer are calculated for analyzing the electronic structure property and stable mechanism of structures. As shown in Figure 5, the P4/mmm-AgN_2_ at 100 GPa and P1-AgN_7_ at 50 GPa are the semiconductor phases with the band gaps of 1.0 eV and 2.4 eV, respectively. For the P4/mmm-AgN_2_, the electronic states of valence bands near the Fermi level are mainly contributed by the Ag_d and N_p orbitals, while the conduction bands near the Fermi level are mainly contributed by the Ag_s and N_p orbitals. For the P1-AgN_7_, the electronic states of valence bands near the Fermi level are mainly contributed by the Ag_d and N_p orbitals, while the conduction bands near the Fermi level are mainly contributed by the N_p orbitals. The P-1-AgN_7_ at 150 GPa is the metal phase, for which the electronic states near the Fermi level are mainly contributed by the N_p orbitals. For the presented band structure and DOS in Figure 6, the P-1-AgN_4_ and P-1-AgN_8_ at 150 GPa are both the metal phases, for which the electronic states of valence bands near the Fermi level are mainly contributed by the Ag_d and N_p orbitals, while the conduction bands near the Fermi level are mainly contributed by the N_p orbitals.

**Figure 7:**
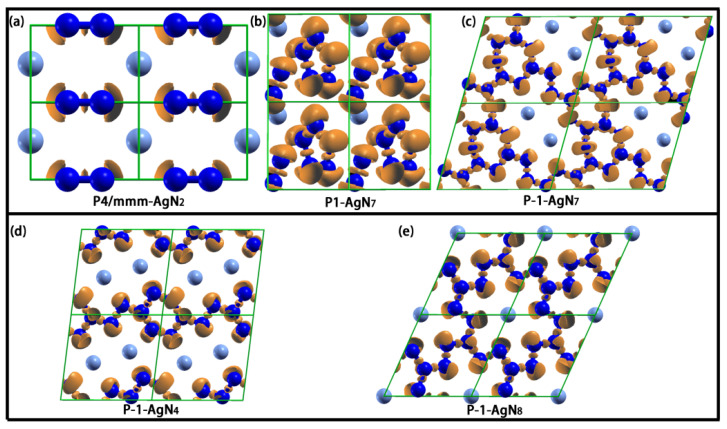
The ELFs of P4/mmm-AgN_2_ (**a**), P1-AgN_7_ (**b**), P-1-AgN_7_ (**c**), P-1-AgN_4_ (**d**) and P-1-AgN_8_ (**e**) (isovalue = 0.8).

In Figure 7, as the fixed value of isovalue (0.8) in ELF, the high localization electronic states between the nitrogen atoms indicate the strong N–N covalent bond interaction. The lone electron pairs distribute at the outside corner of N atoms for reducing the repulsive interaction. In combination with the analysis of Figure 4, we know that the N atom in the dumbbell-shaped N_2_ structure of P4/mmm-AgN_2_ and P1-AgN_7_ hybridizes in the sp state, which is formed by one N–N σ bond and one lone pair electron. The N atom in the N_5_ ring hybridizes in the sp^2^ state, which is formed by two N–N σ bonds and one lone electron pair. In the P-1-AgN_7_, the N_1_, N_3_, N_5_ and N_7_ atoms hybridize in sp^2^ states, which are formed by two N–N σ bonds and one lone electron pair, while the N_2_, N_4_, and N_6_ atoms hybridize in sp^3^ states, which are formed by three N–N σ bonds and one lone electron pair. In the P $\bar{1}$-AgN_4_, the N_1_, N_3_, and N_4_ atoms hybridize in sp^2^ states, which are formed by two N–N σ bonds and one lone electron pair, while the N_2_ atom hybridizes in the sp^3^ state, which is formed by three N–N σ bonds and one lone electron pair. In the P $\bar{1}$-AgN_8_, the N_2_, N_3_, and N_4_ atoms hybridize in sp^2^ states, which are formed by two N–N σ bonds and one lone electron pair, while the N_1_ atom hybridizes in the sp^3^ state, which is formed by three N–N σ bonds and one lone electron pair. No localization electron is distributed around the Ag atom and between the Ag and N atoms due to the weak Ag–N electronic overlap interaction. As the presented charge transfer in Table 2, we can see that the Ag and N atoms are severally the electron donor and receptor, which means the weak Ag–N ionic bond interaction. Clearly, this charge transfer enhances the N–N covalent bond and Ag–N ionic bond interaction, which improves the structural stability. According to the above discussion, we know that the stable mechanism of our predicted Ag–N compounds originates from the strong N–N covalent bond interaction and the weak Ag–N ionic bond interaction. Moreover, the charge transfer between the Ag and N atoms plays an important part in their structural stability.

The energy densities and detonation properties of P-1-AgN_7_ and P-1-AgN_8_ are presented in Table 3. It can be seen that the energy density of P-1-AgN_7_ and P-1-AgN_8_ is 3.9 kJ/g, which is close to that of the TNT (4.3 kJ/g). The detonation velocities of P-1-AgN_7_ (13.58 km/s) and P-1-AgN_8_ (17.59 km/s) are 2.0 and 2.5 times the value (6.90 km/s) of TNT, respectively. The detonation pressures of P-1-AgN_7_ (115.5 GPa) and P-1-AgN_8_ (210.7 GPa) are 6 and 11 times the value (19.00 GPa) of TNT. Thus, the P-1-AgN_7_ and P-1-AgN_8_ are potential candidates for new high-energy density species.

## 4. Conclusions

The crystal structure, electronic structure and energy property of silver nitrides in nitrogen-rich aspects are studied by using the first-principles calculations combining the particle-swarm structural searching. In addition to the reported P-1-AgN_3_, three stable high-pressure phases (P4/mmm-AgN_2_, P1-AgN_7_ and P-1-AgN_7_) and two metastable high-pressure phases (P-1-AgN_4_ and P-1-AgN_8_) are proposed by the structural prediction method. The stable pressure range of P4/mmm-AgN_2_, P1-AgN_7_ and P-1-AgN_7_ are proposed by the enthalpy difference analysis. Interestingly, the novel N_7_ rings and N_20_ rings are firstly found in the folded layer structure of P-1-AgN_7_. In electronic structure analysis, the P4/mmm-AgN_2_ and P1-AgN_7_ are the semiconductor phases, while the P-1-AgN_7_, P-1-AgN_4_ and P-1-AgN_8_ are the metal phases. The analysis of ELF and Bader charge shows that the stable mechanism of predicted Ag–N compounds originates from the strong N–N covalent bond interaction and the weak Ag–N ionic bond interaction. Moreover, the charge transfer between the Ag and N atoms plays an important role for their structural stability. The P-1-AgN_7_ and P-1-AgN_8_ with the high energy densities and excellent detonation properties are potential candidates for new high-energy density species. This work not only enriched the high-pressure phase diagram of Ag–N compounds but also proposed two new high-energy density structures.

## Figures and Tables

**Table 1:** The elastic constants Cij (GPa) of the Ag–N compounds at high pressure.

AgN_x_	(GPa)	C_11_	C_22_	C_33_	C_44_	C_55_	C_66_	C_12_	C_13_	C_15_	C_23_	C_25_	C_35_	C_46_
P4/mmm-AgN_2_	100	563	-	648	85	-	22	313	310	-	-	-	-	-
P1-AgN_7_	50	215	294	231	70	68	42	160	154	−3	173	−5	8	24
P-1-AgN_7_	150	943	942	800	221	207	279	513	396	−85	377	−113	−19	−85
P-1-AgN_4_	150	910	912	710	127	148	270	593	354	−42	338	−39	49	27
P-1-AgN_8_	150	1163	1114	861	193	56	250	604	152	−17	228	−24	12	0.24

**Table 2:** The Bader charge analysis of P4/mmm-AgN_2_, P1-AgN_7_, P-1-AgN_7_, P-1-AgN_4_ and P-1-AgN_8_ at different pressures. The negative and positive values mean the donor and the receptor of charge, respectively.

Structures	Pressure	Element	σ(e)/Atom
P4/mmm-AgN_2_	100	Ag	−0.70
		N	+0.35
P1-AgN_7_	50	Ag	−0.82
		N	+0.117
P-1-AgN_7_	150	Ag	−0.623
		N	+0.089
P-1-AgN_4_	150	Ag	−0.64
		N	+0.16
P-1-AgN_8_	150	Ag	−0.76
		N	0.095

**Table 3:** The energy density and detonation properties of P-1-AgN_7_ and P-1-AgN_8_ compounds in comparison to TNT.

Compounds	Energy Densities (kJ/g)	Detonation Velocity (km/s)	Detonation Pressure (GPa)
P-1-AgN_7_	3.90	13.58	115.5
P-1-AgN_8_	3.90	17.59	210.7
TNT	4.30	6.90	19.00

## Data Availability

The data presented in this study are available on request from the corresponding author.
